# Detection of *Bacillus cereus sensu lato* Isolates Posing Potential Health Risks in Mexican Chili Powder †

**DOI:** 10.3390/microorganisms9112226

**Published:** 2021-10-26

**Authors:** Andrea Guadalupe Celestino Hernández, Vannessa Gómez Ortiz, Jackeline Lizzeta Arvizu Gómez, Miguel Ángel Ramos López, José Alberto Rodríguez Morales, Antonio Flores Macías, Erika Álvarez Hidalgo, Jorge Nuñez Ramírez, Francisco Javier Flores Gallardo, María Carlota García Gutiérrez, Sergio Romero Gómez, George H. Jones, José Luis Hernández Flores, Juan Campos Guillén

**Affiliations:** 1 Facultad de Ciencias Naturales, Universidad Autónoma de Querétaro, Av. de las Ciencias S/N, Querétaro 76220, Mexico; acelestino28@alumnos.uaq.mx (A.G.C.H.); vgomez21@alumnos.uaq.mx (V.G.O.); 2 Unidad Académica de Agricultura, Universidad Autónoma de Nayarit, Carretera Tepic-Compostela, Xalisco CP 63780, Mexico; jackeline.arvizu@uan.edu.mx; 3 Facultad de Química, Universidad Autónoma de Querétaro, Cerro de las Campanas S/N, Querétaro 76010, Mexico; miguel.angel.ramos@uaq.mx (M.Á.R.L.); erika.beatriz.alvarez@uaq.mx (E.Á.H.); jorge.nunez@uaq.mx (J.N.R.); fflores18@alumnos.uaq.mx (F.J.F.G.); maria.carlota.garcia@uaq.edu.mx (M.C.G.G.); sergio.dejesus.romero@uaq.mx (S.R.G.); 4 Facultad de Ingeniería, Universidad Autónoma de Querétaro, Cerro de las Campanas S/N, Querétaro 76010, Mexico; jose.alberto.rodriguez@uaq.mx; 5 Departamento de Producción Agrícola y Animal, Universidad Autónoma Metropolitana, Unidad Xochimilco, Calzada del Hueso 1100, Villa Quietud, Coyoacán, Ciudad de México CP 04960, Mexico; aforesm@correo.xoc.uam.mx; 6 Department of Biology, Emory University, Atlanta, GA 30322, USA; ghjones@emory.edu; 7 Centro de Investigación y de Estudios Avanzados del IPN, Irapuato CP 36824, Mexico

**Keywords:** *Bacillus cereus sensu lato*, tRNA^Cys^-PCR, antibiotics resistance, Mexican chili powder

## Abstract

The potential presence of spore-forming bacteria related to the *Bacillus cereus* group in Mexican chili powder elaborated from *Capsicum annuum* L. is of commercial and clinical interest, because chili powder is an essential spice in the Mexican diet and in diets around the globe. To facilitate detection and isolation of members of this group of spore-forming bacteria from Mexican chili powder samples, we identified colonies that grew on agar medium selective for *Bacillus cereus sensu lato,* supplemented with polymyxin B (10 µg/mL) and ampicillin (10 to 100 µg/mL). The presumptive *B. cereus* (*s.l.*) isolates were tested using a tRNA^Cys^-PCR-based approach and the results identified species related phylogenetically to *B. cereus*, *B. thuringiensis,* and *B. toyonensis*. Their toxigenic potential was assessed by serological tests to detect enterotoxins (Nhe and Hbl) and by PCR targeting the hemolysin BL (*hbl*) component C (*hblC*) and non-hemolytic enterotoxin component A (*nheA*). The antibiotic profiles of the isolates showed a high resistance to β-lactams (100% of the isolates), trimethoprim-sulfamethoxazole (100%), tetracycline (90%), erythromycin (77%), clindamycin (74%), and chloramphenicol (42%). Our results indicate the presence of *B. cereus s.l.* with toxigenic characteristics in Mexican chili powder. Because of the potential for these organisms to cause disease through their production of various toxins, and resistance to antibiotics, we recommend that a microbiological risk assessment must be considered in the Mexican regulatory requirements.

## 1. Introduction

Within the Solanaceae family, pepper (*Capsicum annuum* L.) is one of the most economical and agriculturally important plants cultivated all over the world [1,2]. After China, with 17.5 million tons, Mexico is the second largest producer of fresh pepper with 2.7 million tons at 150,000 hectares cultivated annually [2]. Its importance is based on its nutritional content, diverse bioactive compounds, pungency, aroma, and health benefits for the consumers [1,3]. Additionally, diverse genetic lines of *Capsicum annuum* L. have been developed to produce carotenoids with high commercial value in powdered form as spices or as colorants on agro-food, cosmetics, and products from pharma industries [1,2,3,4,5].

In Mexico, chili powder is used as a spice in diverse seasoned foods, fast food, beverages, snacks, fruits, grains, regional spicy candies, and diverse sauces. Thus, chili powder is an important spice in the Mexican diet [2,6]. However, chili powder is produced from sun-dried peppers, and this production process increases the risk of microbial contamination of the product [2]. In some cases, industrial dryers are used to reduce drying time [2]; however, retention of important traits in chili powder is dependent on the drying procedure. For example, color deterioration in chili powder is greatly influenced by moisture content, storage, temperature, atmospheric conditions, and light [1,7]. Thus, drying chili at high temperatures can reduce the volatile compounds, nutrients, and color content in chili powder. All of these conditions during chili powder production are favorable for microbial contamination and demand a comprehensive microbiological risk assessment, with a focus on potential pathogens.

Among the approaches used for the characterization of microbial load in chili powder from various countries are the determination of total aerobic mesophilic bacteria, aerobic spore-forming bacteria, *B. cereus* detection, and determination of members of the Enterobacteriaceae, yeast, and molds [8,9,10,11,12,13,14,15]. Other studies have been conducted for pathogenic species detection, such as *Bacillus cereus*, *Salmonella* spp., *Clostridium perfringens,* or *Escherichia coli* in paprika powder [8,9,10,11,12,13,14,15]. A documented outbreak of human salmonellosis was traced to paprika powdered potato chips as the main vehicle of transmission in Germany when paprika powder imported from South America was used to flavor the product [16]. It is noteworthy that a molecular approach using 16S rRNA gene sequencing of bacteria, isolated from paprika powder, produced in different countries, identified spore-forming bacteria, facilitating the association of a particular species with its geographical origin. This study was limited, however, by the number of bacterial isolates that were examined [12].

In other studies, several *B. cereus sensu lato (s.l.)* strains have been identified as opportunistic pathogens in chili powder, paprika, and other spices of different geographical origins, and several different toxins have been associated with these strains, viz. cereulide, cytotoxin K, hemolysin BL (HBL), and non-hemolytic enterotoxin (NHE) [14,15]. Toxins from these bacteria have been associated with the diarrheal type of *B. cereus* food poisoning, which is typically characterized by abdominal pain and watery diarrhea [17,18,19,20,21]. In addition to these toxigenic characteristics, *B. cereus s.l.* strains are naturally resistant to penicillin and other β-lactam antibiotics because of their content of β-lactamases [13,14,15,22,23], and some studies reveal that resistance may be extended to other commonly used antibiotics, such as chloramphenicol, gentamicin, imipenem, erythromycin, tetracycline, and the trimethoprim/sulfamethoxazole combination [13,14,15,23]. With these genetic characteristics present in *B. cereus s.l.*, its detection is essential in controlling the spread of potential pathogens present on Mexican chili powder.

Unfortunately, knowledge about the presence of the pathogen *B. cereus* in Mexican chili powder is limited and of equal or greater concern is the lack of phylogenetic information on the microbes that may be present as contaminants in the chili powder. To address these deficiencies in our knowledge, we used standard and molecular microbial procedures on Mexican chili powder samples elaborated from *Capsicum annuum* L., and extended our tRNA^Cys^-PCR method previously reported [24] to detect the presence of antibiotic-resistant *B. cereus s.l.* isolates. Further, we examined the capability of the identified microbes to produce enterotoxins (Nhe and Hbl).

## 2. Materials and Methods

### 2.1. Ampicillin-Resistant Detection of B. cereus s.l.

In a first effort to obtain knowledge about the presence of *B. cereus s.l.* in chili powder elaborated from *Capsicum annuum* L., we selected four chili powder varieties, commercially available and economically competitive in a local market in Queretaro, Mexico. Sample A was a red-hot chili powder used as spice principally for fruit, grains, and beverages. Sample B was a red chili powder used principally as a spice for fast food. Sample C was a red extra hot chili powder used as a spice principally for fruit and grains, and sample D was a red chili powder used for its color content in snacks. Each replicate of 10 g of a sample was homogenized in 90 mL of peptone (0.1% *w*/*v*; Difco Laboratories; Detroit, MI, USA) and treated at 80 °C for 10 min to select endospore-forming bacteria. Serial dilutions were then inoculated into triplicated tryptic soy agar (TSA) medium (Difco Laboratories; Detroit, MI, USA) and incubated at 37 °C during 24 h. Numbers of spore-forming mesophilic bacteria (SMB) from each sample were determined. For the isolation of *B. cereus s.l*., 0.1 mL of the first dilution was inoculated into triplicated agar plates containing *B. cereus* agar base (Sigma-Aldrich, CDMX, México) supplemented with 100 mL/L of Egg Yolk Emulsion and 10 µg/mL of Polymyxin B. To select colonies of *B. cereus s.l*. with ampicillin-resistance, the medium was supplemented at concentrations of 5, 10, 15, 20, 25, 50, 75, and 100 µg/ mL of ampicillin. Plates were incubated at 37 °C for 24 h and observed for growth. The theoretical limit of detection (LOD) was therefore 100 cfu/g. Numbers below this limit (˂100 cfu/g) in our results mean that bacterial growth was not detected in these conditions. Suspected *B. cereus s.l.* colonies that were typically mannitol-negative and lecithinase-positive (zone of precipitation around colonies) were selected, and their identities were confirmed in the same culture medium. *B. cereus* ATCC 10876 was used as reference strains for phenotypic tests and phylogenetic analysis.

### 2.2. tRNA^Cys^ Region Amplification Conditions

**Figure 1:**
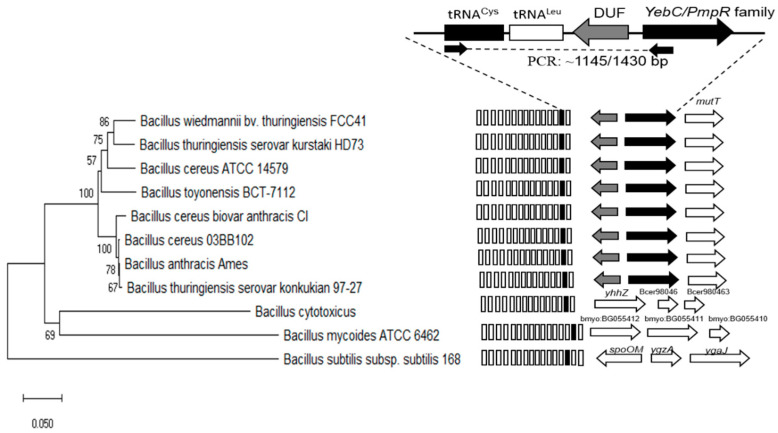
Schematic representation of tRNA^Cys^-PCR strategy for the *B. cereus* group. The cluster of 15 to 17 tRNA genes and the orientation of three specific genes located downstream of tRNA^Leu^ is shown. The primers indicated by the arrows were predicted to yield products of 1145 and 1430 bp, respectively (see Methodology). Phylogenetic analysis from these regions were obtained in MEGA X, using the neighbor-joining method. *B. subtilis* subsp. *subtilis* 168 was designated as the outgroup taxon.

To facilitate detection of suspected *B. cereus s.l.* colonies and *B. cereus* ATCC 10876 as control, we used our tRNA^Cys^-PCR method previously reported [24]. PCR primers were designed to amplify the tRNA^Cys^ region in the *B. anthracis/cereus/thuringiensis/toyonensis/ wiedmannii* group. Primer 1517 (5′-GGCGGCATAGCCAAGTGGTAAGGC-3′) was designed to target the tRNA^Cys^ gene, while primers 1518 (5′-GCTGCCACATAAATTTCACGCCC-3′) and 1520 (5′-GCTACAGAACCGTTCACACCC-3′) were designed to target the yebC/pmpR-like gene. Primers were synthesized by T4OLIGO, Guanajuato, Mexico. The primers were predicted to yield products of 1145 and 1430 bp, respectively (see Figure 1). Amplicons were sequenced using the platform at Macrogen Inc. (Seoul, Korea). Gene sequences were analyzed using MEGA X using the neighbor-joining method [25,26,27,28] and compared with sequences representative of the *B. cereus* group, by a BLAST search, using the GenBank database (http://www.ncbi.nlm.nih.gov, accessed on 16 august 2021).

### 2.3. Duopath^®^ Test for Detection of Nhe and Hbl Bacterial TOXIN Formation

We used the Duopath^®^ Cereus Enterotoxins test (Merck) to detect the non-hemolytic enterotoxin (Nhe) and hemolysin BL (HBL) [29] and tests were performed according to the manufacturer’s specifications. The ampicillin-resistant isolates were streaked onto agar plates containing *B. cereus* agar base (Sigma-Aldrich) supplemented with 100 mL/L of egg yolk emulsion and 100 µg/ mL of polymyxin B. After incubation at 37 °C for 24 h, the colonies were picked and suspended in 1 mL of casein hydrolysate-glucose-yeast extract broth with the following composition (g/L): casein hydrolysate 20.0 (MCD Lab, Mexico), yeast extract 6.0 (Difco, CDMX, México), ammonium sulfate 2.0 (J.T.Baker, CDMX, México), tri-sodium citrate 1.0 (USB Corporation USA), di-potassium hydrogen phosphate 14.0 (J.T.Baker), potassium dihydrogen phosphate 6.0 (J.T.Baker), magnesium sulfate 2.0 (J.T.Baker), and supplemented with 1% glucose (MEYER Lab, CDMX, México). After 5 h incubation at 37 °C, and cooling to room temperature, a 150 μL aliquot of the enrichment was placed into the circular sample port on the Duopath^®^ Cereus Enterotoxins test (Merck) and incubated at room temperature and the interpretation was following manufacturer´s specifications. All strains were evaluated for the presence of representative enterotoxin genes (*nheA* and *hblC*) with primers designed, as well as PCR conditions reported previously [24,30].

### 2.4. Antibiotic Susceptibility Testing

The antibiotic susceptibility of each strain was tested by triplicate using the disc diffusion method determined by the criteria of the European Committee on Antimicrobial Susceptibility Testing (EUCAST) guidelines; criteria were adopted from *Staphylococcus* spp. and *Bacillus* spp. [31]. The bacterial cultures were incubated in liquid soya medium with shaking at 37 °C until the suspension reached an optical density (OD) between 0.4 and 0.5 at 600 nm. A total volume of 100 µL of each strain was spread on Mueller Hinton agar (Bioxon). A total of 18 different antibiotic discs (Oxoid) containing the antibiotics amikacin (AMK 30 μg), ampicillin (AMP 10 μg), carbenicillin (CAB 100 µg), cefalotin (CFT 30 µg), cefotaxime (CTX 30 μg), chloramphenicol (CHL 30 μg), ciprofloxacin (CIP 5 μg), clindamycin (CDM 30 µg), dicloxacillin (DCX 1 µg), erythromycin (ERY 15 μg), gentamicin (GEN 10 μg), netilmicin (NET 30 µg), nitrofurantoin (NTF 300 µg), norfloxacin (NFX 10 µg), penicillin (PEN 10 μg), tetracycline (TET 30 μg), trimethoprim-sulfamethoxazole (SXT 25 μg), and vancomycin (VCM 30 µg) were used for susceptibility testing. After overnight incubation, the mean of diameter in mm of the inhibitory or clear zones around the disc was recorded, and these were interpreted as susceptible, susceptible increased exposure or resistant, according to EUCAST guidelines.

## 3. Results

### 3.1. SMB and B. cereus s.l. Determination

The content of spore-forming mesophilic bacteria (SMB) and presumptive *B. cereus s.l*. counts from four chili powder samples were determined, as described in Methodology. As shown in Table 1, sample B showed the lowest SMB content with 8.0 × 10^2^ cfu/g, followed by sample A with 1.73 × 10^3^ cfu/g, while sample C and D counts were 2.92 × 10^5^ and 3.24 × 10^5^ cfu/g, respectively. These results document the presence of spore-forming bacteria in the tested chili powder samples, validating the need for the next step, detecting the presence of *B. cereus s.l*. The literature indicates that *B. cereus s.l*. is typically resistant to β-lactam antibiotics [13,14,15,22,23]. Accordingly, we used various concentrations of ampicillin in the culture medium during selection with two purposes, first to eliminate ampicillin-susceptible bacteria and second, to facilitate detection and isolation of ampicillin-resistant *B. cereus s.l*. Table 1 shows the presumptive *B. cereus s.l*. counts according with the phenotypical characteristics mentioned in Methodology. The lowest presumptive *B. cereus s.l.* counts were observed in sample B, with the limit of detection (LOD) of 100 cfu/g in absence of ampicillin, while in the presence of different concentrations of ampicillin, no presumptive *B. cereus s.l*. could be detected (LOD = 100 cfu/g). Sample A had 200 cfu/g in the absence of ampicillin, while in the presence of ampicillin (5 to 50 µg/mL), 100 cfu/g was detected. At 75 and 100 µg/mL of ampicillin, no presumptive *B. cereus s.l*. could be detected (LOD = 100 cfu/g). In samples C and D, the counts were 600 and 700 cfu/g, respectively, in the absence of ampicillin. In the presence of ampicillin, presumptive *B. cereus s.l*. in sample C could be detected at a concentration of 5 to 25 µg/mL of ampicillin, with an average of 580 cfu/g. In Sample D, presumptive *B. cereus s.l*. counts averaged 470 cfu/g at concentrations of 5 to 75 µg/mL of ampicillin. From these results, a total of 30 presumptive ampicillin-resistant *B. cereus s.l*. colonies from all chili powder samples were selected, and the properties described above were confirmed in the same culture medium.

### 3.2. Phylogenetic Analysis

For the confirmation of the identities of these ampicillin-resistant presumptive *B. cereus s.l*. colonies, we used the tRNA^Cys^-PCR strategy previously published [24]. In Figure 1, we show that the tRNA^Cys^ gene in *B. cereus s.l*. is part of cluster of 15 to 17 tRNA genes localized downstream of a ribosomal RNA operon. For the diagnostic and phylogenetic analysis we used the specific DNA region between tRNA^Cys^ and *yebC*/*pmpR*-like gene (which encodes a probable transcriptional regulatory protein) located downstream of a gene sequence of unknown function (DUF gene), indicated in Figure 1. The phylogenetic analysis show that this DNA region is specific for *B. anthracis*, *B. cereus s.s*., *B thuringiensis*, *B. toyonensis,* and *B. wiedmannii* related species, while *B. mycoides* and *B. cytotoxicus* show a different gene organization. Therefore, our PCR test is a suitable approach for verification of the identities of the chili powder isolates.

From a total of 30 presumptive ampicillin-resistant *B. cereus s.l.* colonies selected (6 colonies from sample A, 3 colonies from sample B, 4 colonies from sample C, and 17 colonies from sample D), 25 (83 %) were positive for the tRNA^Cys^-PCR product (data not shown), when the primers 1517 and 1518 were used as the first option (~1145 bp PCR product). A negative result for tRNA^Cys^-PCR was obtained for five colonies from sample D, and could be due to a small nucleotide variation in the *yebC*/*pmpR*-like gene region, from which the 1518 primer was designed. Therefore, a new primer, designated 1520, was designed, beginning 285 nucleotides downstream of the DNA region, targeted by the 1518 primer. The tRNA^Cys^-PCR results (~1430 bp PCR product) were positive (data not shown) with this last primer designed for the rest of presumptive ampicillin-resistant *B. cereus s.l*. colonies (17%).

**Figure 2:**
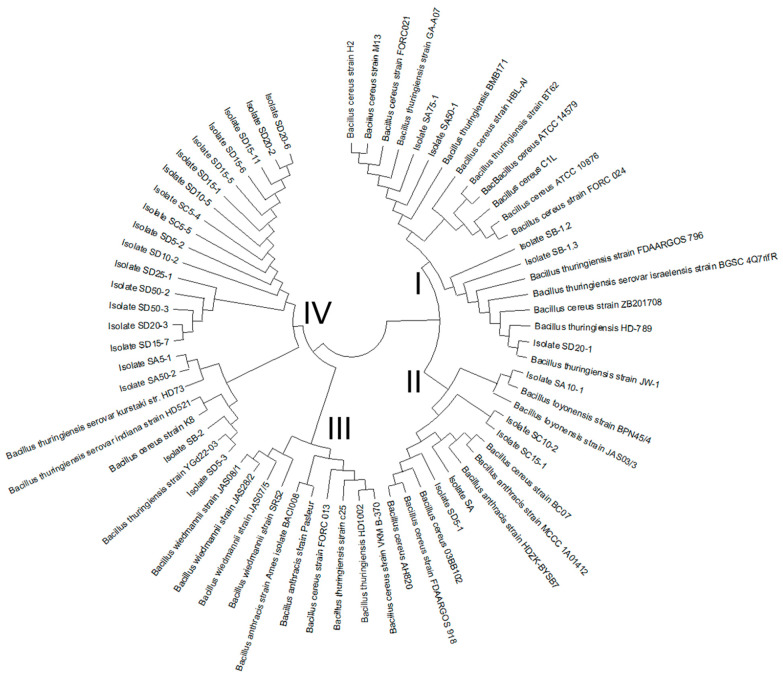
Phylogenetic analysis for tRNA^Cys^-*yebC*/*pmpR* region. The sequences of the isolates obtained from chili powder samples were compared with sequences of representative strains of *B. cereus* group. The isolated colonies obtained clustered within groups I, II, and IV, respectively (see Table 2 for correspondence of each isolate).

Our phylogenetic analysis confirmed the results obtained by tRNA^Cys^-PCR; the presumptive ampicillin-resistant *B. cereus s.l*. colonies are members of the *B. cereus* group. Clustering of the tRNA^Cys^- *yebC*/*pmpR* genes sequences revealed four major species groups (Figure 2). The species isolated from chili powder samples were clustered in Groups I, II, and IV, with the majority of isolates related phylogenetically to *B. cereus* and *B. thuringiensis,* and the isolate SA10-1 closely related to *B. toyonensis* strains from various environmental sources.

### 3.3. Toxigenic Characteristics

**Figure 3:**
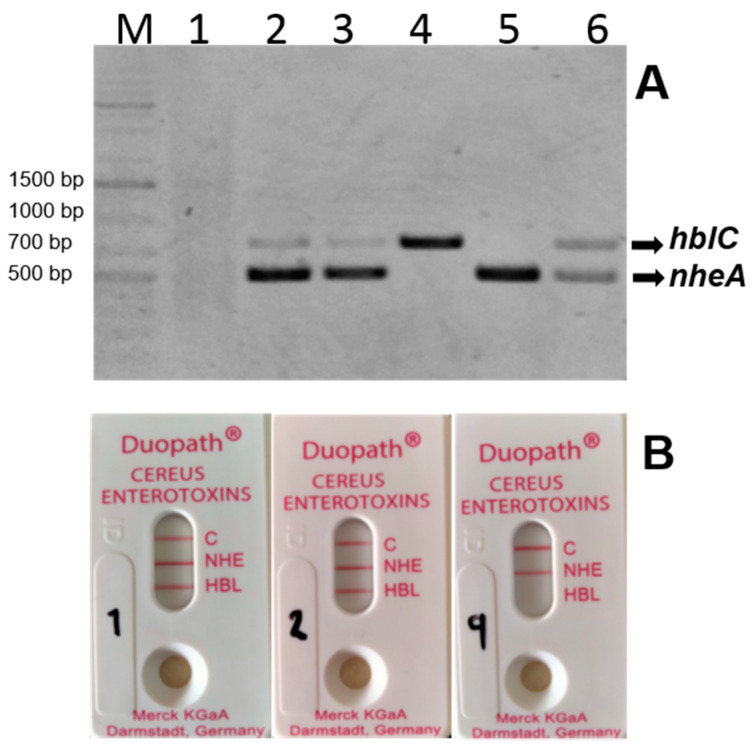
Representative results for toxigenic characteristics. Panel (**A**) shows a representative electrophoresis gel for multiplex PCR. Lane M contains a Thermo Scientific GeneRuler 1 Kb DNA ladder, lane 1; strain SA5-1, lane 2; strain SA, lane 3; strain SB-2, lane 4, 5, and 6 is the control (*B. cereus* ATCC 10876) with single or multiplex PCR respectively. The arrows show the PCR amplification product for hblC (750 bp) and nheA (500 bp) toxin genes. Panel (**B**) shows a representative Duopath test for detection of toxin production indicated by a red line in both the test (Nhe and/or Hbl) and control zones (C). From left to right; *B. cereus* ATCC 10876 (1), SA75-1 (2), and SD20-1 (9).

The toxigenic potential for the isolates was investigated by PCR for *hblC* and *nheA*, as representative genes. Figure 3, panel A shows a representative PCR for *hblC* and *nheA* genes. Lane 1 shows the negative PCR for the strain SA5-1, while lanes 2 and 3 are PCR multiplex positive for the strain SA and SB-2. Lanes 4, 5, and 6 show the PCR positive control using the strain *B. cereus* ATCC 10876 with single or multiplex PCR, respectively. The PCR results are summarized in Table 2. With the exception of one strain (SA5-1) all isolates were positive for at least two toxin genes (97%). The Duopath test for detection of toxin production showed a positive result for the Hbl toxin (Table 2) in 28 strains (90%), including the *B. cereus* ATCC 10876, while the negative strains were SA, SC10-2, and SD20-1. The Nhe toxin was detected in all strains using the Duopath test, including strain SA5-1, even though the PCR result for this strain was negative. In Figure 3B, we show a representative Duopath test for detection of toxin production (NHE and HBL), the results show from left to right production of both toxins for the strain *B. cereus* ATCC 10876 and SA75-1, while for the strain SD20-1, only the NHE toxin was produced. The toxin production capabilities reflected the potential health risk of *B. cereus s.l*. present in Mexican chili powder samples.

### 3.4. Antibiotic Resistance

**Figure 4:**
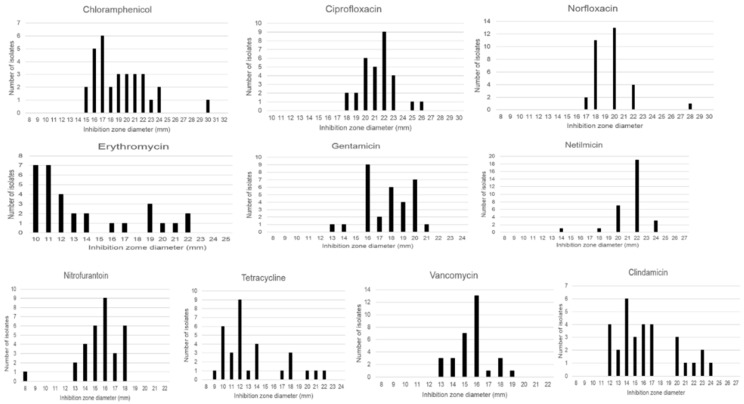
Determination of inhibition zone diameter in the antibiotic disc diffusion tests. Interpretation is based on the EUCAST breakpoints; isolates categorized as susceptible to norfloxacin (S ≥ 17 mm) can be reported as “susceptible increased exposure” to ciprofloxacin (R ˂ 21 mm, S ≥ 50 mm) (see Table 2), vancomycin (R ˂ 10 mm, S ≥ 10 mm), netilmicin (R ˂ 18 mm, S ≥ 18 mm), nitrofurantoin (R ˂ 13 mm, S ≥ 13 mm), gentamicin (R ˂ 18 mm, S ≥ 18 mm), chloramphenicol (R ˂ 18 mm, S ≥ 18 mm), clindamycin (R ˂ 19 mm, S ≥ 22 mm), erythromycin (R ˂ 18 mm, S ≥ 21 mm), and tetracycline (R ˂ 19 mm, S ≥ 22 mm).

The detection and isolation of ampicillin-resistant *B. cereus s.l*. colonies confirmed the following results. All *B. cereus s.l*. isolates, including the *B. cereus* ATCC 10876, were significantly resistant to the tested concentrations of the β-lactam antibiotics penicillin G (PEN), ampicillin (AMP), carbenicillin (CAB), cefalotin (CFT), cefotaxime (CTX), and dicloxacillin (DCX) (Table 2, and see Methodology). All strains were also significantly resistant to the tested concentrations of trimethoprim-sulfamethoxazole (SXT). Our interpretation based on the EUCAST breakpoints, the results in Table 2 and Figure 4 show that the *B. cereus s.l.* isolates were resistant to tetracycline (TET) (90% of the isolates), erythromycin (ERY) (77%), clindamycin (CDM) (74%), and chloramphenicol (CHL) (42%). A lower percentage of strains showed resistance to amikacin (AMK) (6%), gentamicin (GEN) (6%), netilmicin (NET) (3%), and nitrofurantoin (NTF) (3%). On the other hand, *B. cereus s.l*. isolates were susceptible to ciprofloxacin (CIP) (100%), norfloxacin (NFX) (100%), vancomycin (VCM) (100%), netilmicin (NET) (97%), nitrofurantoin (NTF) (97%), amikacin (AMK) (94%), gentamicin (GEN) (94%), chloramphenicol (CHL) (58%), clindamycin (CDM) (26%), erythromycin (ERY) (23%), and tetracycline (TET) (10%).

## 4. Discussion

In Mexico, the cultivated pepper (*Capsicum* spp.) is a phenotypically diverse species grown throughout the country, in different geographical areas, and using various cultivation methods [2,6]. The peppers are used to produce a wide range of chili powders that are subsequently used in a variety of foodstuffs. One potential issue, from a biosecurity perspective, is the possibility that chili powder might be exposed to microbial contamination during its production, storage, commercialization, or when combined with other ingredients to prepare seasoned foodstuffs. Therefore, quality and safety must be important considerations in certifying chili powder for use, with the specific focus on detection of potential pathogens to avoid the spread of food-borne illnesses in consumers.

With these considerations in mind, the studies presented here demonstrate the presence in chili powder samples of levels of aerobic-mesophilic bacterial spores of 8.0 × 10^2^ to 3.24 × 10^5^ cfu/g and bacterial species closely related phylogenetically to the *B. cereus s.l.* group, and in low quantities (˂700 cfu/g). With regard to this results, in some reports, *B. cereus s.l.* levels of ˂10^4^ cfu/g have been found in red chili powder from India [32], in paprika and pepper (˂80 cfu/g) in Germany [14], and in chili powder and organic paprika (˂23 cfu/g) in the USA [15]. The European Food Safety Authority (EFSA) has noted that some reports of food-borne disease outbreaks caused by diverse *B. cereus* isolates have been associated with bacterial concentrations above 10^5^ cfu/g foodstuff, but in a few reports, concentrations of 10^3^ cfu/g [33] were involved in reported outbreaks.

The studies reported are also significant in that they demonstrate the presence of enterotoxin genes (*hblC* and *nheA*) in the *B. cereus s.l.* isolates. The PCR results were confirmed and extended by the Duopath test for detection of toxin production, which showed the presence of Hbl and Nhe toxins in 90% and 100% of isolates, respectively. The presence of these toxins increases the potential for these strains to cause food-borne illnesses in the consumers of chili powder. Other reports on studies involving paprika, chili powder, and other seasonings have also shown that the *nhe* and *hbl* gene sequences and toxin production are detected at high frequency [14,15]. Taking into account the available information and in agreement with our results, the levels of *B. cereus s.l.* found in Mexican chili powder are low and probably not represent a health risk according to EFSA. However, the results are a minimum representative sample from a wide chili powder diversity in Mexico; thus, requiring additional efforts to extend similar microbial analysis throughout the country, where chili powder is produced.

Generally, chili powder is a ready-to-eat spice in different foodstuffs in Mexico, and for the consumers, the inherent health risk occasioned by the presence of *B. cereus s.l.* could be increased due to the antibiotic resistance properties of these organisms. That resistance could easily compromise treatment procedures in cases of infections caused by these toxigenic bacteria after consumption of chili powder. Thus, obtaining the *B. cereus* antibiotic resistance profile is highly relevant to public health. Our results in this context are in concordance with other reports where *B. cereus s.l.* has been shown to be resistant to β-lactam antibiotics resulting from the presence in their genomes of genes coding for β-lactamase enzymes [13,14,15,22,23]. It is worth mentioning that *B. cereus s.l.* isolates were also significantly resistant to trimethoprim-sulfamethoxazole, tetracycline, erythromycin, clindamycin, and chloramphenicol. Similar antibiotic resistances have been reported in the USA, Europe, and China [14,23,34]. Similar to the results of other reports, our *B. cereus s.l.* isolates were generally susceptible to other antibiotics, including ciprofloxacin, norfloxacin, vancomycin, netilmicin, nitrofurantoin, amikacin, and gentamicin [14,23,34]. A few *B. cereus s.l.* isolates, however, showed reduced susceptibility to chloramphenicol, clindamycin, erythromycin, and tetracycline in our studies. Although the number is limited, there are studies that indicate that vancomycin is the antibiotic of choice for *B. cereus* infections [23,34,35]. The results obtained in this study confirm the need for further studies of such resistance breakpoints.

Additional insight into the taxonomic and toxigenic relationship of *B. cereus s.l.* isolates obtained in this study is necessary by comparative genomics, as well as by other methods of molecular and biochemical discrimination analyses necessary to understand its metabolic capabilities and close relationship among members of the *B. cereus* group.

## 5. Conclusions

The results obtained in this study show that Mexican chili powder can be contaminated with *B. cereus s.l.* strains, which represent a potential health risk because of their antibiotic resistance and their production of biological toxins. Furthermore, chili powder has a long shelf life and the spore-forming ability of *B. cereus s.l.* allows it to persist for long periods. All of these considerations would seem to support the development of appropriate monitoring programs for microbial quality to assess the distribution of microorganisms in seasonings, such as chili powder, and the potential risk to public health.

## Figures and Tables

**Table 1:** Spore-forming mesophilic bacteria (SMB) and presumptive *B. cereus s.l*. counts in Mexican chili powder samples.

Sample	SMB ^a^	BC ^b^ (0)	BC (5)	BC (10)	BC (15)	BC (20)	BC (25)	BC (50)	BC (75)	BC (100)
A	1.73 × 10^3^	200	100	100	100	100	100	100	˂100	˂100
B	8.0 × 10^2^	100	˂100	˂100	˂100	˂100	˂100	˂100	˂100	˂100
C	2.92 × 10^5^	600	633	600	666	566	433	˂100	˂100	˂100
D	3.24 × 10^5^	700	600	433	633	533	533	333	233	˂100

^a^ Spore-forming mesophilic bacteria (SMB) counts. ^b^ Presumptive *Bacillus cereus s.l*. (BC) counts. To select colonies with ampicillin-resistance, the medium was supplemented at concentrations of 5, 10, 15, 20, 25, 50, 75, and 100 µg/ mL of ampicillin, indicated by numbers in parentheses. The theoretical limit of detection (LOD) was therefore 100 cfu/g. Numbers below this limit (˂100 cfu/g) in our results mean that bacterial growth was not detected in these conditions.

**Table 2:** Toxigenic and antibiotics characteristics for *B. cereus s.l* isolates from Mexican chili powder samples. Presence (+) or Absence (−) for toxin production and toxin genes.

Strain	Chili Powder Sample	Toxin Production		Toxin Genes		Antibiotics		Susceptible Increased Exposure
		Hbl	Nhe	*hblC*	*nheA*	Resistance	Susceptible	
*B. cereus* ATCC 10876	−	+	+	+	+	AMP, CAB, CFT, CTX, DCX, PEN, SXT, CDM	AMK, CHL, CIP, GEN, NET, NTF, NFX, TET, VCM	ERY
SA	A	−	+	+	+	AMP, CAB, CFT, CTX, DCX, ERY, PEN, SXT, TET	AMK, CHL, CIP, CDM, GEN, NET, NTF, NFX, VCM	
SA5-1	A	+	+	−	−	AMP, CAB, CFT, CTX, DCX, PEN, SXT, CHL, TET	AMK, CIP, GEN, NET, NTF, NFX, VCM	ERY, CDM
SA10-1	A	+	+	+	+	AMP, CAB, CFT, CTX, DCX, ERY, PEN, SXT	AMK, CHL, CIP, CDM, GEN, NET, NTF, NFX, TET, VCM	
SA50-1	A	+	+	+	+	AMP, CAB, CFT, CTX, DCX, PEN, TET, SXT	AMK, CHL, CIP, ERY, GEN, NET, NTF, NFX, VCM	CDM
SA50-2	A	+	+	+	+	AMP, CAB, CFT, CTX, DCX, PEN, TET, SXT, CHL	AMK, ERY, GEN, NET, NTF, NFX, VCM	CIP, CDM
SA75-1	A	+	+	+	+	AMP, CAB, CFT, CTX, DCX, NTF, PEN, TET, SXT	AMK, CHL, CIP, CDM, ERY, GEN, NET, NFX, VCM	
SB-2	B	+	+	+	+	AMP, CAB, CFT, CTX, DCX, ERY, PEN, SXT, TET, CDM	AMK, CHL, NET, NTF, NFX, VCM	CIP, GEN
SB-1.2	B	+	+	+	+	AMP, CAB, CFT, CTX, DCX, ERY, PEN, SXT, TET, CDM	AMK, CHL, NET, NTF, NFX, VCM	CIP, GEN
SB-1.3	B	+	+	+	+	AMP, CAB, CFT, CTX, DCX, ERY, PEN, SXT, TET, CDM	AMK, CHL, NET, NTF, NFX, VCM	CIP, GEN
SC5-4	C	+	+	+	+	AMP, CAB, CFT, CTX, DCX, ERY, PEN, TET, SXT, CDM	AMK, CHL, CIP, NET, NTF, NFX, VCM	GEN
SC5-5	C	+	+	+	+	AMP, CAB, CFT, CTX, DCX, ERY, PEN, TET, SXT, CDM	AMK, CHL, CIP, NET, NTF, NFX, VCM	GEN
SC10-2	C	−	+	+	+	AMP, CAB, CFT, CTX, DCX, ERY, PEN, SXT, TET, CDM	AMK, CHL, NET, NTF, NFX, VCM	CIP, GEN
SC15-1	C	+	+	+	+	AMP, CAB, CFT, CTX, DCX, ERY, PEN, SXT, CDM	AMK, CHL, CIP, GEN, NET, NTF, NFX, TET, VCM	
SD5-1	D	+	+	+	+	AMP, CAB, CFT, CTX, DCX, ERY, PEN, TET, SXT, CDM	AMK, CHL, CIP, NET, NTF, NFX, VCM	GEN
SD5-2	D	+	+	+	+	AMP, CAB, CFT, CTX, DCX, ERY, PEN, TET, SXT, CHL, CDM	AMK, CIP, NET, NTF, NFX, VCM	GEN
SD5-3	D	+	+	+	+	AMP, CAB, CFT, CTX, DCX, PEN, SXT, CHL, TET, CDM	AMK, CIP, NET, NTF, NFX, VCM	GEN, ERY
SD10-2	D	+	+	+	+	AMP, CAB, CFT, CTX, DCX, ERY, PEN, TET, SXT, CHL, CDM	AMK, CIP, GEN, NET, NTF, NFX, VCM	
SD10-5	D	+	+	+	+	AMP, CAB, CFT, CTX, DCX, ERY, PEN, TET, SXT, CHL, CDM	AMK, GEN, NET, NTF, NFX, VCM	CIP
SD15-1	D	+	+	+	+	AMP, CAB, CFT, CTX, DCX, ERY, PEN, TET, SXT, CHL	AMK, CIP, CDM, GEN, NET, NTF, NFX, VCM	
SD15-5	D	+	+	+	+	AMP, CAB, CFT, CTX, DCX, ERY, PEN, TET, SXT, CHL, CDM	AMK, CIP, GEN, NET, NTF, NFX, VCM	
SD15-6	D	+	+	+	+	AMP, CAB, CFT, CTX, DCX, ERY, PEN, TET, SXT, CDM	AMK, CHL, CIP, NET, NTF, NFX, VCM	GEN
SD15-7	D	+	+	+	+	AMK, AMP, CAB, CFT, CTX, DCX, ERY, NET, PEN, TET, SXT, CHL, GEN, CDM	CIP, NTF, NFX, VCM	
SD15-11	D	+	+	+	+	AMK, AMP, CAB, CFT, CTX, DCX, ERY, PEN, TET, SXT, CDM	CHL, GEN, NET, NTF, NFX, VCM	CIP
SD20-1	D	−	+	+	+	AMP, CAB, CFT, CTX, DCX, PEN, TET, SXT, CDM	AMK, CHL, CIP, GEN, NET, NTF, NFX, VCM	ERY
SD20-2	D	+	+	+	+	AMP, CAB, CFT, CTX, DCX, ERY, PEN, TET, SXT, CHL, CDM	AMK, NET, NTF, NFX, VCM, GEN	CIP
SD20-3	D	+	+	+	+	AMP, CAB, CFT, CTX, DCX, ERY, PEN, TET, SXT, CHL, CDM	AMK, NET, NTF, NFX, VCM	CIP, GEN
SD20-6	D	+	+	+	+	AMP, CAB, CFT, CTX, DCX, ERY, PEN, TET, SXT	AMK, CHL, CIP, GEN, NET, NTF, NFX, VCM	CDM
SD25-1	D	+	+	+	+	AMP, CAB, CFT, CTX, DCX, ERY, PEN, TET, SXT, GEN, CDM	AMK, CHL, NET, NTF, NFX, VCM	CIP,
SD50-2	D	+	+	+	+	AMP, CAB, CFT, CTX, DCX, ERY, PEN, TET, SXT, CHL, CDM	AMK, CIP, NET, NTF, NFX, VCM	GEN
SD50-3	D	+	+	+	+	AMP, CAB, CFT, CTX, DCX, ERY, PEN, TET, SXT, CHL, CDM	AMK, CIP, NET, NTF, NFX, VCM, GEN	
